# Impact of Hierarchical Cation-Exchange Membranes’ Chemistry and Crosslinking Level on Electrodialysis Demineralization Performances of a Complex Food Solution

**DOI:** 10.3390/membranes13010107

**Published:** 2023-01-13

**Authors:** Elodie Khetsomphou, Francesco Deboli, Mateusz L. Donten, Laurent Bazinet

**Affiliations:** 1 Institute of Nutrition and Functional Foods (INAF), Dairy Science and Technology Research Centre (STELA) and Department of Food Sciences, Université Laval, Quebec, QC G1V 0A6, Canada; 2 Laboratoire de Transformation Alimentaire et Procédés ÉlectroMembranaires (LTAPEM, Laboratory of Food Processing and ElectroMembrane Processes), Université Laval, Quebec, QC G1V 0A6, Canada; 3 Department of Chemical Engineering, KU Leuven, 3001 Leuven, Belgium; 4 Amer-Sil S.A., 8281 Kehlen, Luxembourg

**Keywords:** electrodialysis, complex food system, whey demineralization, composite cation-exchange membrane, hierarchical cation-exchange membrane

## Abstract

Hierarchical cation-exchange membranes (hCEMs) fabricated by blade coating and UV crosslinking of ionomer on top of a porous substrate demonstrated promising results in performing NaCl demineralization. In the food industry, complex solutions are used and hCEMs were never investigated before for these food applications. The performances of two different coating chemistries (urethane acrylate based: UL, and acrylic acid based: EbS) and three crosslinking degrees (UL5, UL6, UL7 for UL formulations, and EbS-1, EbS-2, EbS-3 for EbS formulations) were formulated. The impacts of hCEMs properties and crosslinking density on whey demineralization performances by electrodialysis (ED) were evaluated and compared to CMX, a high performing CEM for whey demineralization by ED. The crosslinking density had an impact on the hCEMs area specific resistance, and on the ionic conductance for EbS membrane. However, 70% demineralization of 18% whey solution was reached for the first time for hCEMs without any fouling observed, and with comparable performances to the CMX benchmark. Although some properties were impacted by the crosslinking density, the global performances in ED (limiting current, demineralization duration, global system resistance, energy consumption, current efficiency) for EbS and UL6 membranes were similar to the CMX benchmark. These promising results suggest the possible application of these hCEMs (UL6, EbS-2, and EbS-3) for whey demineralization by ED and more generally complex products as an alternative in the food industry.

## 1. Introduction

Electrodialysis (ED), first developed for water and wastewater treatment, is one of the membrane-based technologies that is finding increased use in the food industry. ED is an electrically-driven process used for the concentration, separation, and purification of solutions. Indeed, the food industry mainly adopted ED to improve process performances and food quality in traditional food products’ preparation, to find new products and processes designed to meet the food requirements related to health and nutrition, as well as to satisfy the demands of changing regulations related to by-product treatment and waste in food processes [1,2]. Furthermore, the low energy consumption of ED, its modular design, efficiency, and ease of use, as well as the heat sensitivity of many food products are among the reasons for its rapid growth [3]. Therefore, in the dairy industry, ED is largely used to reduce the salt content in whey, which is the major by-product of cheesemaking. Whey is a complex food system composed of water, lactose, raw proteins, lactic acid, fat, and salts. Its high salt content leads to nutritional imbalance and taste impairment, which becomes an issue when using whey in food formulations; therefore, whey must be desalted to become a valuable solution, to be implemented in a variety of products [2,4]. In addition to being economically wise, whey has a high biological oxygen demand which prevents its discharge as wastewater into sewage, making its re-utilization necessary.

ED is based on selective ion transport through ion-exchange membranes (IEMs) [2,5,6]. In addition, IEMs segregate a solution regarding to the charge of the species it contains; therefore, the effectiveness of the process mostly depends on IEMs’ selectivity and conductivity [7]. However, those membranes are often highly engineered materials that come at a high price. Indeed, membranes represent 25–30% of the total cost of a fully automated ED unit [2]. Advances in membrane technology, especially in novel materials, can make this technology even more competitive. IEMs can be classified into homogeneous or heterogeneous materials, depending on their physical structure and fabrication method [8]. However, the use of homogeneous membranes can be limited by their cost compared to the heterogeneous IEMs by interfering with the large-scale implementation in smaller value applications [9]. To reduce the cost of IEMs, development of heterogeneous membranes was implemented. Not only was the production cost reduced, but the mechanical stability of the membranes was also improved [10]. However, this improvement came at the cost of a lower selectivity and conductivity compared to homogeneous membranes.

To combine the desirable transport properties of homogeneous materials with the stability and simple fabrication of heterogeneous membranes, novel hierarchically structured anion-exchange membranes have been recently developed [11,12]. Hierarchical ion-exchange membranes (hIEMs) are composed of a highly porous substrate coated with a thin layer of ion-exchange polymer. This concept allows for decoupling the mechanical and transport properties of the membrane individually. Moreover, this approach enables limiting the thickness of the ionomer layer (well below 50 μm), which favors the conductivity and enables material savings on the ionomer precursors [11,12]. Recently, it has been reported that cation-exchange coatings of hierarchical exchange membranes (hCEMs) can be fabricated using UV curable formulations containing urethane acrylate oligomers and monomers [13], or using UV-initiated radical polymerization of formulations containing acrylic oligomers and monomers [14]. To fabricate the hCEMs, the reactive formulations were applied as a thin film on top of a PVC-SiO_2_ porous substrate and crosslinked in situ in a UV-initiated radical curing process. The high-volume porosity and the exposed silica grains on the substrate’s surface enhancing the adhesion, make PVC-SiO_2_ the ideal substrate for these types of membranes [15]. Two different chemistries were used to fabricate the hIEMs, and the performances of these membranes on model salt solutions desalination by ED have been reported [13,14]. Furthermore, these membranes were compared to heterogeneous membranes which are recognized as less efficient than homogeneous ones. In addition, no results can be found on the performances of these membranes on actual industrial solution conditions and on the impact of these chemistries on the ED process with more complex solutions, such as food solutions.

Moreover, one of the main limitations of IEMs, especially in the food industry, is related to fouling issues which lead to significant alterations in the membranes. An undesirable evolution with time in the initial properties can be observed, thus decreasing the overall process performance [16,17,18,19]. Different strategies have been applied to reduce these phenomena, such as cleaning protocols [20,21,22]. Therefore, IEMs used for a food application also need to withstand the harsh conditions of the cleaning process.

In this context, this paper presents the characterization of two different hCEMs coating chemistries. Three coating formulations with different crosslinking density were evaluated for each coating chemistry. This paper aims: (1) To demonstrate their applicability for demineralization of complex solutions, such as the ones in the food industry, i.e., whey (model food solution), (2) to evaluate whey demineralization performance of an ED stack composed of these hierarchical ion-exchange membranes, (3) to study the impact of the different chemistries and ratios of monomers and oligomers on membrane performances, and 4) to compare these performances with those of the highest performing industrial food grade homogeneous cation-exchange membranes.

## 2. Materials and Methods

### 2.1. Materials

Two sets of hCEMs with two different coating chemistries were fabricated and provided by Amer-Sil S.A. (Kehlen, Luxembourg). The first set of hCEMs, UL chemistry, consists of an urethane acrylate resin functionalized with sulfonic groups, first described by Deboli et al. [13]. The second set of hCEMs, EbS coating chemistry, is an acrylic acid composed of the membranes formulated with the addition of acrylic resins and sulfonated acrylates, also reported by Deboli et al. [14]. The manufacturing process of the hCEMs is detailed in the aforementioned papers. In this study, three different formulations were developed for each chemistry, the ratios are detailed in Table 1. The same ratios of monomers and oligomers from the ones used previously by Deboli et al. were used for EbS formulations. For UL formulations, the same ratios were used for UL5 and UL6 as Deboli et al., whereas different ratios were used for UL7.

Food grade Neosepta ion-exchange membranes: Cation-exchange membranes (CMX), used as a benchmark, and anion-exchange membranes (AMX) were purchased from Astom (Tokyo, Japan).

Sweet whey powder (lot: 1019003001) was provided by Parmalat (Victoriaville, QC, Canada). All experiments were carried out using the same lot of whey powder. The average concentrations of lactose (68%), protein (11%), and moisture (5%) of the powder were obtained from the manufacturer, while the ash content and the cation composition (7%—cations: K^+^ (55.6%), Na^+^ (19.9%), Ca^2+^ (19.9%), and Mg^2+^ (4.6%)) were determined for this study.

### 2.2. Methods

#### 2.2.1. Membrane Characterization and Morphology

Prior to testing in an ED cell, the membranes were characterized in terms of physicochemical properties.

##### Thickness

The thickness of hCEMs and the benchmark were measured using an electronic digital micrometer (Marathon Watch Company Ltd., Richmond Hill, ON, Canada). Six measurements were taken at different points of the membrane sample and the reported values consist of the average [23].

##### Microscopy

The fabricated hIEMs were observed under a digital microscope (VHX-7000, Keyence, Osaka, Japan) to assess the continuity and homogeneity of the ion-exchange coating layer. The coating thickness was determined by cross sections of the membranes using digital image analysis (VHX-7000 Ver 1.4.23.17, Keyence, Osaka, Japan).

##### Profilometer

A microstylus surface profilometer (Dektak XT, Bruker Nano Surfaces, Tucson, AZ, USA) with 64-bit parallel processing operation was used to observe the profiles of the membranes. The profilometer provides a three-dimensional topography of the membrane surface. A stylus with a radius of 5 µm was traced across the membrane in hills and valley modes with a vertical measurement range of 524 µm. For each membrane, scans with scan length and map extent of 1000 µm and scan duration of 5 s were performed. The analysis software used was Vision64 (Bruker Nano Surfaces, Tucson, AZ, USA). Then, average roughness (Ra) and average values of the highest heights and deepest alleys (Rz) of the membranes were measured to assess the coating layer topography [23]. For each membrane, the measurements were performed in triplicate on three different samples.

##### Ionic Conductance and Conductivity

Ionic conductance (G) of the membrane was measured in 0.5 M NaCl solution using two electrodes through a plane cell, which was designed by Institut de Chimie et des Matériaux Paris-Est (ICMPE) (Université Paris-Est, Thiais, Val de Marne, France). The cell was used in combination with an YSI conductivity meter (Model 35; Yellow Springs Instrument Co., Yellow Springs, OH, USA) following the procedure described by Bazinet and Araya-Farias [24]. Equilibration of the membrane samples was performed in the measurement electrolyte for 30 min prior to the measurement. The electrical conductance (G) measurement (G = 1/R) can be used to determine the electrical resistance (R). Therefore, the transversal electrical resistance of the membrane, $R_{m}$ (Ω), was calculated as follows:

(1) $$R_{m}=R_{m+s}-R_{s}$$

where $R_{m+s}$ is the overall resistance (of the membrane and the reference solution measured together in Ω) and $R_{s}$ is the resistance of reference solution in Ω.

Finally, specific electrical conductivity (κ, in S·cm^−1^) can be calculated by considering the thickness of membrane using the following equation [25,26]:

(2) $$\kappa =\frac{l}{R_{m}A}$$

where *l* is the thickness of the membrane (in cm), $R_{m}$ is the transversal electrical resistance of the membrane (in Ω), and A is the measurement area (1 cm^2^ in the present measurement setup). Six different measurements at various points of the studied sample were taken and averaged to obtain the reported values.

##### Selectivity

A laboratory handmade setup of two cell compartments equipped with a saturated calomel electrodes in each half cell was used to measure the membrane potential as previously reported by Deboli et al. [13]. The membrane (active measurement area of 19.63 cm^2^) was installed between two half cells. One half cell was filled with a 0.5 M KCl solution, whereas in the second half cell, a KCl solution with a concentration of 0.1 M was used. A potentiostat (Origaflex 01A, Origalys, Rillieux-La-Pape, France) monitored the membrane potential until a stable value was reached. The membrane selectivity was calculated as the ratio of the experimental potential ($\Delta V_{exp}$) and the theoretical potential for a 100% selective membrane ($\Delta V_{th}$) calculated from the Nernst equation:

(3) $$\alpha =\frac{\Delta V_{exp}}{\Delta V_{th}}\times 100$$

A theoretical membrane potential ($\Delta V_{th}$) of 36.68 mV was used for the calculation [13].

##### Area Specific Resistance

Plane area specific resistance (ASR) of the membrane was measured in a 0.5 M NaCl solution as previously detailed in [13]. Equilibration of the membranes was carried out for at least 48 h prior to the measurements in the electrolyte. ASR was determined using a lab made four-electrode cell with an active area of 19.63 cm^2^ controlled by a potentiostat (Origalflex 01A, Origalys, Rillieux-la-Pape, France). Polarization curves were recorded (current sweep from 0 to 0.5 A, scan rate 10 mA s^−1^) and the Ohmic region of the plots was used to extrapolate the resistance value. The ASR of the membrane was determined as the difference of the resistance values with and without the membrane according to the following equation:

(4) $$ASR=\left(R_{m}-R_{0}\right)\cdot \;A$$

where $R_{m}$ and $R_{0}$ are the cell resistance (in Ω) with and without the membrane, respectively, and A is the active measurement area (in cm^2^ in this setup).

#### 2.2.2. Electrodialysis experiments

##### ED Setup and Configuration

**Figure 1:**
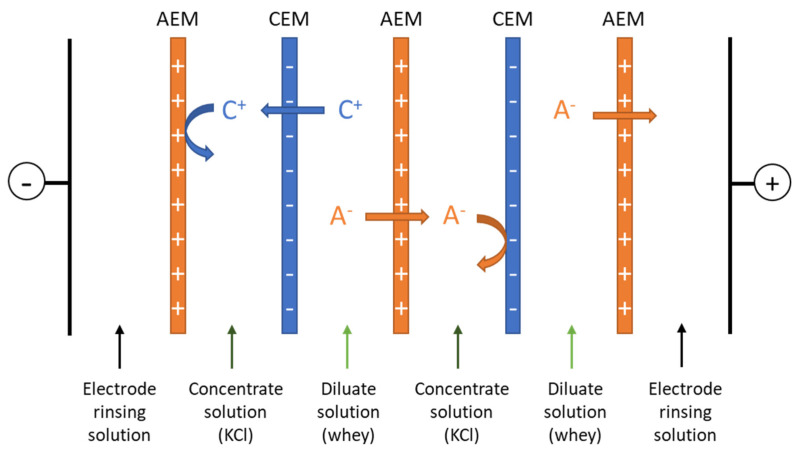
Configuration of the electrodialysis cell used for whey demineralization. C^+^, cation; A^−^, anion; AEM, anion-exchange membrane; CEM, cation-exchange membrane.

ED experiments were carried out in an ED MP type cell (ElectroCell AB, Täby, Sweden) with an effective membrane surface area of 100 cm^2^. The ED configuration consisted of two hCEMs or benchmark food grade CMX along with three Neosepta food grade anion-exchange membranes (AMX). The stack has been arranged in order to obtain a six-compartment cell configuration as shown in the scheme of Figure 1. The coating side of the formulated hCEMs was orientated toward the diluate compartment following previous experiments by Villeneuve et al. [11]. The membranes were conditioned overnight in 2 g·L^−1^ KCl solution in order to achieve full activation. A stainless-steel electrode cathode and a dimensionally stable electrode (DSA-O_2_) were used for the cathode and anode, respectively. Pumps (Baldor Electric Company, Fort Smith, AR, USA) were used to recirculate the concentrate, diluate, and electrode rinsing solutions in the cell with a flow rate monitored by flowmeters (Blue-White Industries Ltd., San Diego, CA, USA) in order to promote a turbulent flow and prevent polarization phenomena: The linear velocity along the membrane was 0.12 cm.s^−1^. A solution containing 20 g·L^−1^ of Na_2_SO_4_ and 2 g·L^−1^ of KCl was used respectively as an electrode rinsing solution and concentrate in all the experiments, while the diluate consisted of sweet whey.

##### Limiting Current Density

Prior to the ED experiments, the above-mentioned limiting current density of the system in real conditions was evaluated, in order to determine the voltage to be used in this protocol. The voltage values chosen in this study were determined by the related limiting current density experiments carried out using the method of Cowan and Brown [27]. Indeed, of all the membranes, 80% of the lowest limiting current density value obtained was chosen. The related voltage was determined and the obtained value was used to set the voltage for all ED experiments [2,11]. Limiting current density experiments of the system were performed in duplicates. The same membrane samples were used for the repetitions for each membrane type. The system was briefly cleaned with water between two runs and left to rest for 1 h to recover, in order to avoid an impact on the membranes’ integrity as described by Doyen et al. [28]. The ED stack was tested using the aforementioned configuration and the voltage was gradually increased by 0.5 V increments from 0 to 30 V. Then, the current intensity was monitored through the process. The resistance (U/I) as a function of the reciprocal current (1/I) was plotted, and the intersection of the lines represents the limiting current density value [27].

##### Performance Evaluation

pH

The pH of KCl and whey solutions was measured using a pH-meter (Models SympHony SP70P and SympHony SP20, VWR Scientific Products, Radnor, PA, USA).

Conductivity

KCl and whey solution conductivities were measured using a conductivity meter (Model 3100, Yellow Spring Instruments, Yellow Springs, OH, USA) equipped with an automatic temperature compensation (ATC) and an immersion probe (Model 3252, cell constant K = 1 cm^−1^).

Demineralization Rate

The demineralization rate (*DR*) was monitored throughout the experiment as follows:

(5) $$DR=\frac{\kappa_{0}-\kappa_{t}}{\kappa_{0}}$$

where $\kappa_{t}$ and $\kappa_{0}$ are the diluate conductivity at time t (end of the process) and 0, respectively.

Mineral Concentration

Mineral concentration evolution was measured for each run on KCl fractions collected every 15 min during the ED treatments by ICP-OES (Agilent 5110 SVDV Agilent Technologies, Mulgrave, Australia) [11]. The wavelengths used were 393.847; 422.673 nm for calcium, 766.490 nm for potassium, 279.553; 280.270; 285.213 nm for magnesium, 588.995; 589.592 nm for sodium, and 177.434; 178.222; 213.618; 214.914 nm for phosphorus. Flow injection analyzer (Quikchem 8500 serie 2, Zellweger Analytic, Inc., Lachat Instruments Division, Milwaukee, WI, USA) was used for chloride analysis, according to the Quikchem method 10-117-07-1-C: chlorine in water. The analyses of these ions were performed in axial and radial views.

Current Efficiency and Energy Consumption

Current efficiency (*η*) and energy consumption (*EC*) have been evaluated after each experiment according to the following equations [13,29]:

(6) $$\eta =\frac{zFV_{d}\left(C_{t}-C_{0}\right)}{n\int_{0}^{t}I\left(t\right)dt}$$

(7) $$EC=U\int_{0}^{t}I\left(t\right)dt\;$$

where z is the absolute valence of the transported ion, *F* is the Faraday constant, $C_{t}$ and $C_{0}$ are the concentrations of potassium in KCl recovery solution at times t and 0, $V_{d}$ is the KCl solution volume, n is the number of cell pairs, *I* the current, *U* the voltage, and t the process duration.

##### Whey Demineralization

All whey demineralization experiments were performed in triplicates using 600 mL of an 18% whey solution as it is the concentration commonly used and performed in the dairy industry. The ED experiments were carried out in voltage-controlled mode to yield constant electric field strength until 70% whey demineralization was reached. For each membrane type, the same membrane samples were used for the replicates, and after the third experiment, the ED cell was disassembled to evaluate the impact of consecutive runs on the membranes’ properties. Moreover, three consecutive runs enable the ensuring of the presence or not of fouling [30]. Therefore, the ED stack was briefly cleaned with water between the ED runs to avoid the impact of electrolyte and feed solution of one ED run to another ED run. The flow rates were set at 0.7 L·min^−1^ (or 3.7 m s^−1^) for diluate (600 mL) and concentrate (800 mL) compartments, and at 1.0 L·min^−1^ (or 5.3 m s^−1^) for electrode rinsing solution compartments. Experimental parameters, such as pH and conductivity of diluate solution and the current across the ED stack, were recorded every 15 min throughout the experiment. Moreover, the performance of the systems was evaluated in terms of DR, current efficiency, and energy consumption. Furthermore, the thickness and conductivity of each membrane type were analyzed after the three repetitions.

#### 2.2.3. Statistical Analyses

All experiments were performed in triplicates. The data were subjected to one-way analyses of variance (ANOVA), Tukey (among all the membranes), and Dunnett (CMX as the reference) tests at a probability level of 0.05 using SigmaPlot software (version 12.0, Systat Software, San Jose, CA, USA). The thickness and conductivity of each membrane before and after ED testing were compared by *t*-test at a probability level of 0.05. For current-voltage curves, two repetitions were performed, and the data were subjected to ANOVA and Tukey tests at a probability level of 0.05.

## 3. Results and Discussion

### 3.1. Membrane Characterization and Morphology

The average thickness of hCEMs and the respective functional coatings are reported in Table 2. Within the UL membranes, no significant difference could be found in the membrane thickness (0.430 ± 0.014 mm) and the average coating layer thickness (29.2 ± 1.7 µm). For EbS membranes, the membrane thickness was similar for all formulations (0.422 ± 0.007 mm), whereas EbS-2 showed higher coating thickness compared to the other two membranes (53.5 ± 1.8 µm vs. 44.45 ± 0.55 µm, respectively). In general, the thickness of the ionomer layers was found to be higher for EbSs than for ULs. This difference can be explained by the nature of the precursors used: UL ion-exchange layers were formulated with waterborne resins, whereas neither solvent nor water was used in the formulation of the ionomer for EbS. During the curing process, water evaporates from the coating, thus leading to a lower thickness for UL membranes, whereas for EbS formulation, all the precursors are reactive. Overall, a low thickness of the ion-exchange layer is beneficial for the conductivity of the membrane. Indeed, ionomer coatings contribute to a decrease in conductivity of the hCEM [12]; therefore, maintaining low thickness is seen as beneficial for the performance of the membrane. Finally, hCEMs were significantly thicker than the reference (+66%), due to a significant contribution of the substrate.

**Figure 2:**
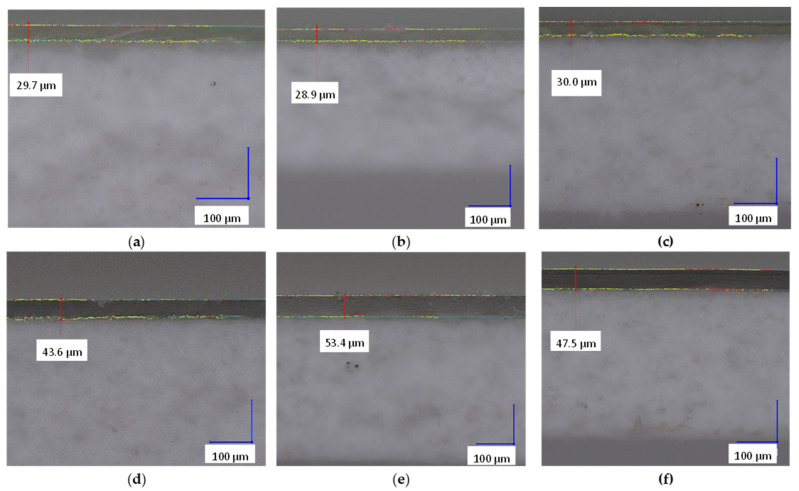
Representative cross-section micrographs for (**a**) UL5, (**b**) UL6, (**c**) UL7, (**d**) EbS-1, (**e**) EbS-2, and (**f**) EbS-3. The images show a clear interface between the substrate and the coating layer.

**Figure 3:**
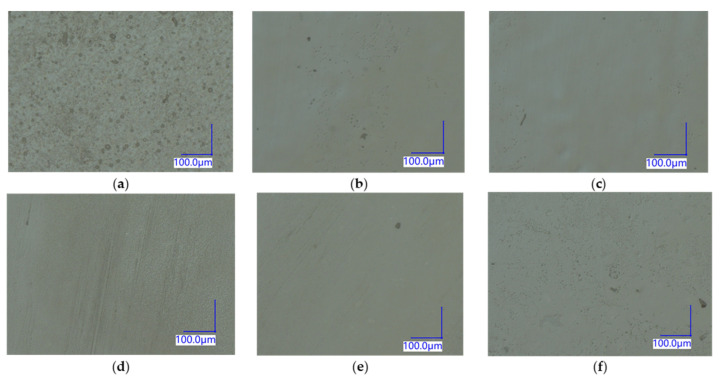
Representative micrographs of the surface for (**a**) UL5, (**b**) UL6, (**c**) UL7, (**d**) EbS-1, (**e**) EbS-2, and (**f**) EbS-3. The micrographs were not taken for the paper samples but can be considered as representative.

Figure 2 shows examples of the cross-section micrographs that were performed to study the structure of the membranes. A sharp interface between the coating layer and the substrate can be observed in all the hCEM samples, demonstrating the layered hierarchical structure of the membranes. The digital optical microscope images (Figure 3) did not show any sign of defects, such as cracks or pits that could hinder the performances of the membranes. These samples can be considered as representative of hCEMs.

Ra and Rz of all the membranes are reported in Table 2. Ra and Rz values were similar for all the membranes with an average of 0.59 ± 0.09 µm and 5.34 ± 1.04 µm, respectively. Due to the homogeneity of the membrane surface, small values of Ra and Rz, and sensitivity of the probe, large standard deviations were observed. However, the deviations are minimal compared to the entire membrane. Similar standard deviations were observed by Kadel et al. [31] for ultrafiltration membranes. A continuous and homogeneous coating surface is crucial for membranes’ transport selectivity.

### 3.2. Ionic Conductance and Conductivity

Table 3 reports the conductance values measured for the fabricated samples. Within the UL membranes, no significant difference could be found with an averaged value of 42.46 ± 0.25 mS. The crosslinking density may not have any influence on this formulation. However, for the EbS membranes, EbS-3 conductance is significantly lower than the other two formulations (33.56 ± 1.61 mS vs. 38.45 ± 0.09 mS, respectively). Its high crosslinking degree causes a decrease in water content leading to a decrease in ion mobility; therefore, a decrease in conductance [7,32]. Moreover, UL type and EbS type membranes exhibited significantly different conductance values: UL type demonstrated higher conductance than EbS type, which may be due to the highly hydrophilic precursors used to formulate UL membranes. Indeed, the resulting UL coating layers are then more hydrophilic which enhance the interaction with water molecules, increasing the conductance. Finally, when comparing the membranes with the reference, the conductance of CMX (46.05 ± 0.27 mS) was fairly higher than the conductance of the UL membranes (+7.4%) and significantly higher than the EbS membranes (+16.5% for EbS-3 and +27.1% for the others).

In terms of conductivity, the conductivities of all UL membranes are similar with an averaged value around 12.12 ± 0.56 mS·cm^−1^. Regarding EbS membranes, EbS-3 conductivity is significantly lower than the other two membranes as expected from the previous values of conductance (4.55 ± 0.45 mS·cm^−1^ vs. 7.86 ± 0.68 mS·cm^−1^, respectively). Compared to the benchmark, hCEMs have an advantage owing to their significantly higher apparent thickness than the reference. Indeed, UL membranes’ conductivity is substantially greater than the CMX (+44%). Moreover, EbS-1 and EbS-2 conductivities are similar to the reference, whereas EbS-3, which showed a really low conductance, also demonstrates poor conductivity (−46%). Furthermore, it was already reported that an increase in the thickness of membranes leads to an increase in conductivity values [33]. These authors suggested that the dependency of the conductivity on membrane thickness may be due to structural changes due to production processes, a difference in the water uptake or a layered structure of the membranes. It is not yet possible to pinpoint which of these effects is predominant or whether there is a synergy among them here.

### 3.3. Area Specific Resistance and Selectivity

Table 3 displays the ASR and selectivity values for the hCEMs and the benchmark. The results show that ASR values decrease with the crosslinking density (UL5: 4.19 ± 0.31 Ω·cm^2^ and UL7: 6.37 ± 0.35 Ω·cm^2^) which also impact the selectivity. Indeed, the selectivity increases with the increasing crosslinking density (UL5: 63% and UL7: 81%). A similar trend can be observed within the EbS membranes (EbS-1: 6.75 ± 0.45 Ω·cm^2^ and EbS-3: 10.53 ± 0.26 Ω·cm^2^). The strong dependency of these properties on the coating’s crosslinking density was previously reported by Deboli et al. The authors found that increasing the crosslinking density of hCEMs allows for improving the selectivity of the membranes, at the cost of a higher resistance. Similar results can be found with the investigated membranes. UL membranes show lower selectivity compared to EbS membranes. The difference observed with EbS membranes can be explained by the lower ASR values. Indeed, it was reported that UL membranes exhibited high water uptake content, especially for the less crosslinked membranes [13]. The higher hydrophilicity of the coating (urethane acrylate chemistry) resulting from the polar groups in the formulation makes them more sensitive to the moisture [34]. Owing to the high water content, the ASR will be reduced but at the expense of lower selectivity. When compared to the commercial membranes CMX (ASR 2.81 Ω·cm^2^—selectivity 91%), UL membranes demonstrated higher ASR with lower selectivity, whereas EbS membranes generally offer a similar selectivity but at a higher resistance level.

### 3.4. Electrodialysis Experiments

#### 3.4.1. Limiting Current Density

Table 4 presents the limiting current density values and the voltage associated with the whole electrodialysis cell for each membrane stacking. Within each chemistry, even though no differences between the membranes were reported (UL: 20.8 ± 0.2 V and EbS: 19.2 ± 1.6 V), the trend seems to indicate that the limiting voltage decreases with the increasing crosslinking density. Moreover, the ANOVA statistical analysis carried out on all the membranes did not reveal any differences among the samples compared to the CMX with an averaged value of 16.7 ± 1.4 mA·cm^−2^ corresponding to a voltage value of 20.0 ± 1.2 V.

As mentioned, during the electrodialysis experiments, the system works in a voltage-controlled mode. The value set corresponded to 80% of the voltage associated with the lowest limiting current density. Therefore, in this case, the lowest limiting current density is EbS-3 (13.9 ± 1.5 mA·cm^−2^) which leads to a fixed voltage for all whey demineralization experiments at U = 13.00 V, in order to compare the membranes in the same anode/cathode voltage difference.

#### 3.4.2. Change in Membrane Properties

Prior to presenting the electrodialysis performances, thicknesses of the membranes are presented in Table 5 and were evaluated before and after three consecutive ED runs. For hCEMs, the statistical analysis showed no significant difference between the membrane thicknesses before electrodialysis and after the experiment with an averaged value of 0.424 ± 0.018 mm. Moreover, after dismantling the system, no fouling was visually observed on all the membranes. Similar observations were performed on the reference with an averaged value of 0.148 ± 0.004 mm.

The conductivities of CMX and hCEMs before and after electrodialysis are presented in Table 5. For UL membranes, the ANOVA statistical analysis carried out on all the membranes shows that only UL5 and UL7 demonstrated a decrease in conductivity after electrodialysis (−9.7% and −11.6%, respectively). For EbS samples, the value obtained after ED is higher for EbS-1 (+24.1%); however, this result is seen as an outlier since the damage of the membrane was noticed in this specific ED. Fragments of the coating were found to be delaminated, likely due to an error in its fabrication process, leaving areas of the porous substrate exposed and leading to an effective increase in conductivity. Overall, the results indicate that there was no significant damage (fouling or scaling) caused to the membranes by the proteins and minerals present in the treated solution. SEM/TEM analyses were not performed to evaluate fouling since fouling phenomena lead to the decrease in membrane conductivity due to (1) the increase in membrane resistance by formation of surface fouling layer or internal membrane fouling and (2) the deposition of fouling agents on membrane ion-exchange groups [16]. This is the reason why conductivity measurement is very sensitive to slight changes. Therefore, a fouled membrane, especially for complex solutions, would show a decrease in its conductivity [24,29,30].

#### 3.4.3. Whey Demineralization

**Figure 4:**
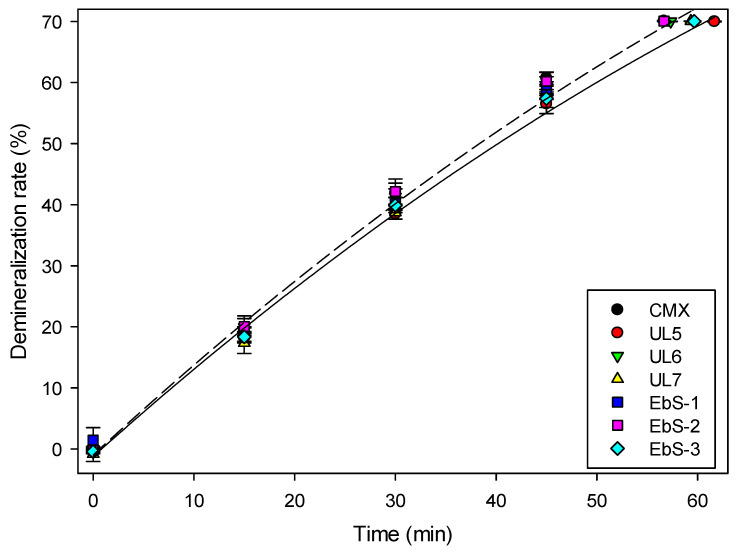
Whey demineralization as a function of time in ED using CMX or hCEMs.

The performances of the hCEMs were tested in an electrodialysis whey demineralization process to investigate the impact of their properties (chemistry and crosslinking). Demineralization rates observed for each membrane as a function of time are presented in Figure 4. Among UL membranes, no significant difference was observed with an averaged demineralization time around 59.4 ± 1.5 min. Similar observations were noticed for EbS membranes with an averaged time to reach 70% demineralization around 57.7 ± 1.3 min. Moreover, all the fabricated membranes showed good demineralization rates compared to the reference—decrease in conductivity in the diluate compartment with time. Interestingly, the time needed to demineralize 70% of the initial solution was found to be similar to the reference for all hCEMs except for UL5; 61.7 min were needed to reach 70% whey demineralization for UL5 compared to 56.7 min for the reference (+8.8%). This slightly higher duration can be explained by (1) the loss of conductivity observed previously (Table 4), (2) the fact that this membrane exhibited one of the highest limiting current densities (Table 4), thus the voltage set may not be the most optimal one for this membrane (under the limiting current density, increasing the voltage increases the mass transfer, thus it may reduce the demineralization duration [2]), and (3) compared to the other UL membranes, this formulation has less functional groups. The transport of ions through IEMs is made possible thanks to the ionic functional groups present in the polymer constituting the membrane. Therefore, for similar chemistry, having a higher number of functional groups allows for a higher transport of ions. Kadel et al. were able to reach 17% DR during a 120-min 18% whey solution demineralization using AEM with a similar fabrication process as the hCEMs, but also for the reference using CMX and AMX [23]. The difference observed for the benchmark is due to the 10 times higher membrane’s surface area used in the cell for the present study.

**Figure 5:**
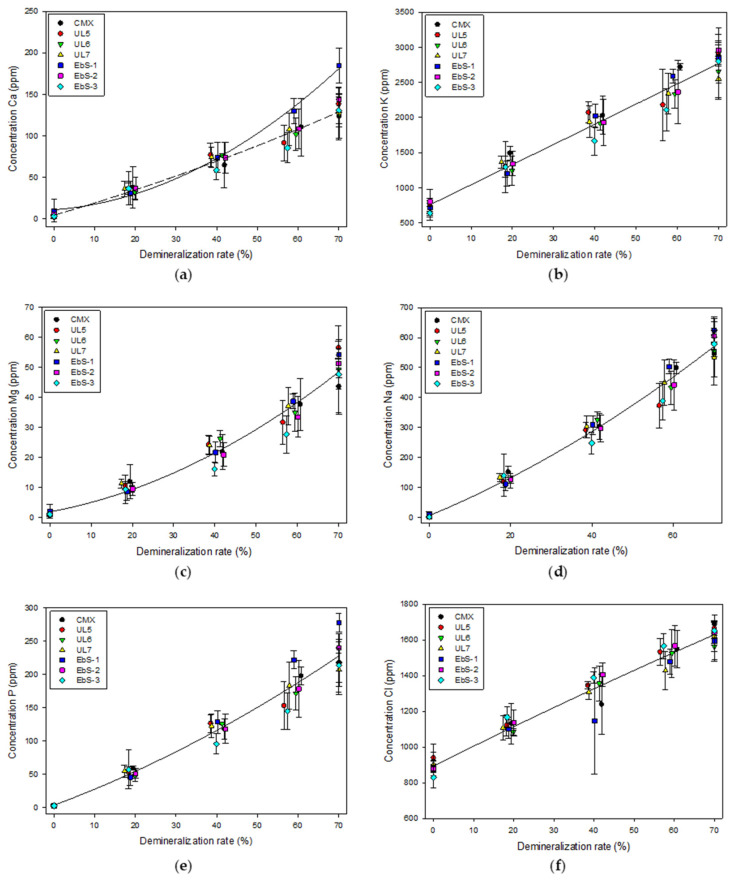
Concentration of ions in the concentrate compartment during electrodialysis as a function of the demineralization rate: (**a**) Calcium; (**b**) potassium; (**c**) magnesium; (**d**) sodium; (**e**) phosphorous; (**f**) chloride.

To confirm the demineralization/mineralization phenomena between the different tested membranes, the concentration of the main ions in the concentrate KCl solution was analyzed. As expected, the concentration of all the ions for all membranes increased during the experiments (Figure 5). However, the migration speed was different according to the species. This is explained by their concentration in the whey solution as well as by their intrinsic characteristic. Potassium ions are the first to migrate as 55.6 ± 1.8% of the cations of the whey solution are composed of potassium. Moreover, monovalent ions are removed faster than divalent ions due to their higher mobilities [35]. Therefore, the concentration of calcium (19.9 ± 0.8% of the cations) is one of the lowest in the solution. Indeed, Aider et al. demonstrated that when the concentration of charged species decreased, their possibility to migrate also decreased [36]. This is due to the greater interactions between the molecules. In addition, Ca^2+^ and Mg^2+^ are the cations with the smallest ionic radii but having the highest hydration numbers compared to the other cations present in the solution, which slow their migration through the membrane due to steric effects. Moreover, it was demonstrated that these cations hold water molecules in their hydration shell very strongly to be detached compared to monovalent ions which explained their slower removal [37]. Furthermore, except for calcium, all the membranes presented the same migration. Therefore, for calcium ions, the concentration in KCl for the cell using EbS-1 was significantly higher than the other membranes at the end of the process (184 ± 21 ppm vs. 132 ± 6 ppm). In the case of EbS-1, the membrane delaminated during the process due to an error in the fabrication of the membrane, leaving some coating parts as free. As a result, calcium ion could move freely through the membrane in the coating-free section regardless of its higher radius and due to its higher electrophoretic mobility. Similar observations could not be seen for magnesium ion. This may be due to the lower initial concentration (4.6 ± 0.2% of the cations) of this ion, which may be very low to detect any significant difference. Overall, these analyses show that mineralization performances for hCEMs are comparable to the reference.

#### 3.4.4. Variation of pH

**Figure 6:**
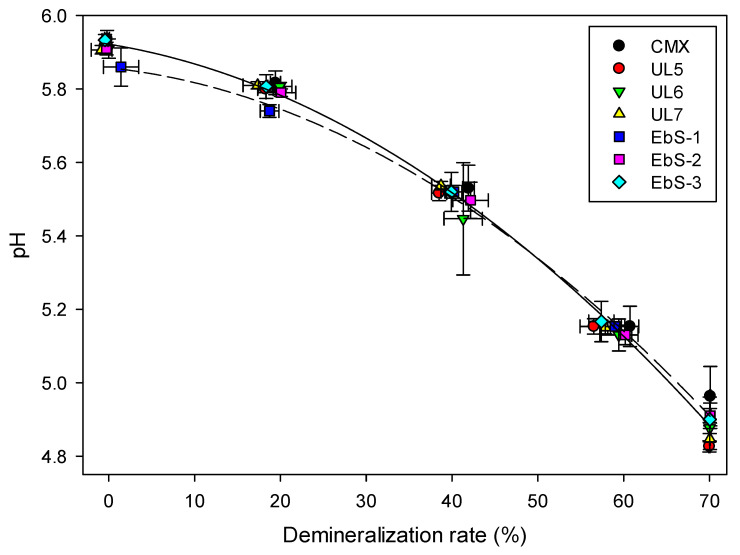
pH evolution of whey during electrodialysis as a function of demineralization rate with hCEMs or CMX.

The pH of the diluate compartment, presented in Figure 6, decreases by 1.0 unit throughout the demineralization process for all treatments and regardless of the membrane type (around 5.9 at the beginning of the process and 4.9 at the end). Indeed, Delbeke et al. first observed a decrease of 0.39 and 1.38 pH units for a 70% and 90% whey demineralization of a 25% whey solution, respectively [38]. Similarly, Villeneuve et al. observed a decrease of 0.25 unit during demineralization of sweet whey for a solution of 6.5% total solid whey [11]. Lemay et al. suggested that this decrease may be caused by the transport of weak acids, such as HPO_4_^2−^ ions, to the concentrate compartment, forcing the dissociation of H_2_PO^4−^ to re-establish the equilibrium [39].

#### 3.4.5. Global System Resistance

**Figure 7:**
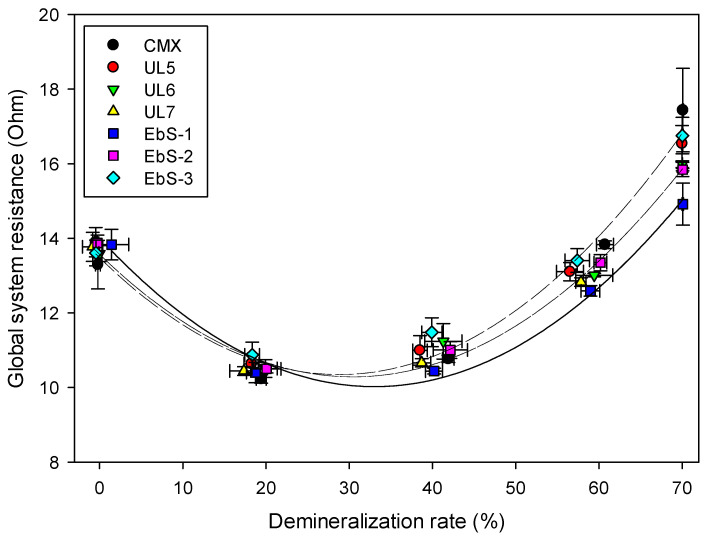
Evolution of global system resistance during whey demineralization as a function of demineralization rate with hIEMs or CMX.

The evolution of the global system resistance is illustrated in Figure 7 as a function of the demineralization rate. The global electrical resistance of the stack for all membranes was initially around 13.71 ± 0.17 Ω. Thereafter, it dropped until reaching a minimum, and then each membrane increased to reach their final global system resistance. The parabola-shaped pattern of the global system resistance could be explained by the coupled impact of conductivities in both diluate and concentrate compartments between 20% and 40%, which also corresponds to the percentage at which the system resistance was at its minimum. When one compartment is more conductive than the other, the least conductive compartment limits the transport of electric charges, thus leading to a higher resistance. When the conductivity of both compartments is equal, there is no more limiting compartment, and the global system resistance is at its minimum. The global internal resistance is linked to the conductivity of the solutions; therefore, the resistance decrease in the first part of the experiment was due to the whey conductivity increase. Then, when both solutions are at the same conductivity, the feed (diluate) solution conductivity becomes the main cause of resistance, and the global resistance starts increasing again, thus the shape pattern is obtained [11].

Regarding the membranes, comparing the difference between the initial (0% DR) and final (70% DR) global system resistance value of UL membranes, no difference was observed for the membranes with an averaged value of 16.13 ± 0.28 Ω. For the EbS membranes, the figure shows that the global system resistance increases with the crosslinking degree. Moreover, the system using EbS-1 demonstrated the lowest final global system resistance (14.92 ± 1.08 Ω) which can be explained by the delamination presented above. The resistance, which is still present, comes from the substrate and the areas that are still covered by the coating. Comparing the initial (0% DR) and final (70% DR) global system resistance value of each membrane, UL6, UL7, and EbS-2 showed significant differences compared to the benchmark (15.99 ± 2.40 Ω, 15.85 ± 2.08 Ω, 15.84 ± 1.96 Ω vs. 17.44 ± 4.13 Ω, respectively), while the global resistance using UL5 and EbS-3 are similar to the reference and significantly higher than the other membranes (16.54 ± 2.65 Ω and 16.75 ± 3.15 Ω, respectively). EbS-3 demonstrated higher membrane’s resistance and ASR considering its high crosslinking degree; the obtained global system resistance compared to the EbS and UL membranes are as expected. For UL5, the higher global system resistance obtained due to the decrease in conductivity observed previously, probably explains the longer demineralization time to reach 70% whey demineralization. Indeed, ion mobility is reduced with the increasing resistance.

#### 3.4.6. Energy Consumption and Current Efficiency

As presented in Table 6, energy consumption was calculated to evaluate the electrodialysis performances. The energy consumption was found to be slightly higher using UL5 and UL7 than CMX (+6.38% and +5.48%). No significant difference was observed in energy consumption using the other hCEMs compared to the CMX. This difference can be explained by the decrease in conductivity observed for both membranes previously as the energy consumption calculation is based on the system resistance influenced by the membranes.

Furthermore, the current efficiency for potassium ion was calculated for all the membranes. Potassium was chosen as it is the most present ion in the solution. As presented in Table 6, the results demonstrate that current efficiencies of the system using the fabricated membranes were similar to the benchmark except for UL7 (44.28% vs. 33.24%). Based on the concentration of potassium, even though no significant difference was observed in the ion concentration by ICP during the mineralization of KCl solution, the concentration of potassium for UL7 was the lowest (2550 ± 290 ppm vs. 2844 ± 78 ppm) at the end of the process, which can explain the lower value acquired. The current efficiencies obtained for all hCEMs used for whey demineralization are lower than those obtained by Francesco et al. [13,14] for NaCl desalination (44.28% vs. 93.42%) due to the complexity of the whey solution. In addition, these observations are as expected since the current efficiency for laboratory cell setups can reach up to 90%, whereas it can only reach up to 50% for industrial installments [40,41,42,43,44,45,46,47]. The setup for this present study is closer to an industrial commercial installment.

### 3.5. Global Performances

**Figure 8:**
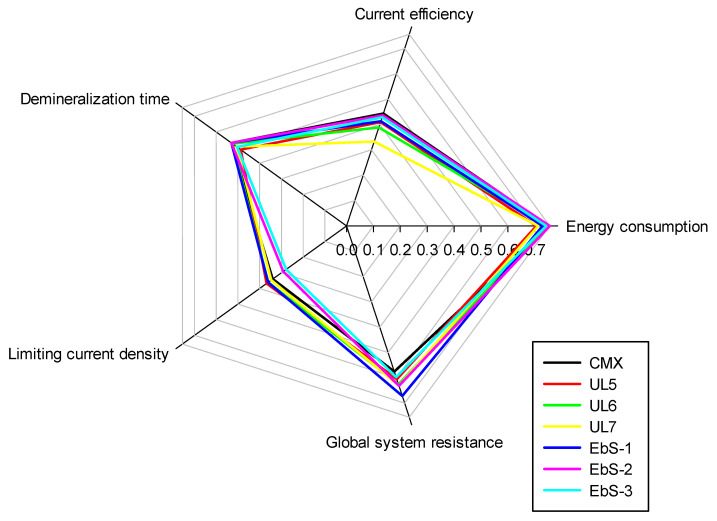
Radar graph of the global ED performance of hIEMs and CMX.

The global performances of the membranes in electrodialysis were compared by plotting radar graphs (Figure 8) and analyzing the area between the samples. Indeed, the results for each parameter were modified to attribute a score. A high score is associated with a better performance; therefore, the highest area is correlated to a better global performance in electrodialysis. For instance, for the energy consumption, a high energy consumption is assimilated to a poor performance; however, for this radar graph, the values were modified by taking the opposite value in order that a high score of energy consumption is equivalent to a good performance. All values were modified to obtain a score in the same range for all parameters.

Concerning the performances of the membranes, due to the delamination issue from the fabrication process, the results for EbS-1 are not representative of its performance. The statistical analysis demonstrated that UL5 and UL7 were significantly different from the CMX. All the other membranes exhibited comparable performances as the commercial reference. These observations are in line with the results obtained.

It is important to note that EbS membranes demonstrated difficulties in handling as a result of the apparent rolling, which was observed after wetting. This is the result of the mismatch between expansion of the coating and substrate upon wetting. In these conditions, the shear stress created when flattening the membrane may cause its breaking, especially when using brittle substrates, such as the PVC-SiO_2_ membrane used in this study.

Finally, Table 7 shows a comparison of the performances of alternative cation-exchange membranes for the same or relatively close dairy product applications reported in the literature.

## 4. Conclusions

The conditions commonly found and/or used in the whey industry for whey demineralization are 18% whey solution—70% demineralization. The hCEMs were tested for the first time for whey demineralization by ED under those conditions. The results provide a convincing proof of applicability of these types of membranes in ED processes, where complex solutions containing salts, lactose, organic acids, and proteins are used. The performances of two different coating chemistries (urethane acrylate based: UL, and acrylic acid based: EbS) and three crosslinking degrees (UL5, UL6, UL7 for UL formulations, and EbS-1, EbS-2, EbS-3 for EbS formulations) were tested and compared to a commercial benchmark membrane. All fabricated membranes successfully demineralized 70% of an 18% whey solution without damages nor fouling observed on the membranes.

UL5 and UL7 were the only membranes to show a decrease in their conductivity after the process. This small decrease resulted in a longer demineralization time, a higher energy consumption, and a poorer current efficiency. Furthermore, UL membranes exhibited the lowest selectivity (UL5—63%; UL6—74%; UL7—81%; CMX—91%) among all the membranes. Compared to EbS membranes, the observed difference may be due to the more hydrophilic nature of the coating layer. This feature can be improved by introducing less polar precursors to reduce the hydrophilicity of the functional coating. All things considered, the better performance of UL6 is likely due to a better tradeoff between conductivity and selectivity resulting from IEC and crosslinking of the ionomer.

EbS membranes demonstrated decreasing conductivities (EbS-1: 8.54 ± 0.72 mS·m^−1^; EbS-2: 7.18 ± 0.45 mS·cm^−1^; EbS-3: 4.55 ± 0.45 mS·cm^−1^) with the increasing crosslinking density. The impact of the crosslinking density is greater than for the UL membranes. Regardless, the resulting global system resistances during ED were lower than or similar to the benchmark. Overall, EbS membranes exhibited similar performance to the commercial reference. Consequently, this study has shown that among all the prepared membranes, UL6, EbS-2, and EbS-3 have the most promising behavior to compete with the commercial CMX for food applications.

Finally, more work should be carried out on the membranes, such as certifying the food grade level of the hCEMs, as well as investigating the impact of the cleaning process used in the dairy industry, in order to ensure that the membranes could be an alternative to the commercial CMX food grade membranes.

## Figures and Tables

**Table 1:** Ion-exchange coating formulation ratios.

**Sample**	**UA02**	**UA06**
UL5	0.12	0.31
UL6	0.00	0.42
UL7	0.00	0.5
**Sample**	**EBE3105**	**EBE830**
EbS-1	0.30	0.15
EbS-2	0.15	0.30
EbS-3	0.00	0.45

UA2 and UA6 are a bi-functional and a hex-functional UV-curable urethane acrylate resin, respectively. EBE3105 and EBE830 are a bi-functional and a hex-functional UV-curable polyester acrylate resin, respectively.

**Table 2:** hIEMs and commercial reference membranes’ thickness, Ra and Rz, and hIEMs coating layer thickness.

Sample	Membrane Thickness (mm)	Coating Thickness (µm)	Ra (µm)	Rz (µm)
CMX	0.145 ± 0.005 ^b^ *	-	0.47 ± 0.15 ^a^	3.22 ± 1.09 ^a^
UL5	0.408 ± 0.007 ^a^	30.1 ± 0.9 ^c^	0.61 ± 0.13 ^a^	7.01 ± 0.92 ^a^
UL6	0.433 ± 0.028 ^a^	26.7 ± 2.6 ^c^	0.64 ± 0.15 ^a^	4.84 ± 0.55 ^a^
UL7	0.448 ± 0.025 ^a^	30.8 ± 0.8 ^c^	0.40 ± 0.15 ^a^	5.37 ± 3.54 ^a^
EbS-1	0.430 ± 0.015 ^a^	45.0 ± 1.8 ^b^	0.67 ± 0.07 ^a^	4.34 ± 0.18 ^a^
EbS-2	0.425 ± 0.005 ^a^	53.5 ± 1.8 ^a^	0.59 ± 0.15 ^a^	5.95 ± 1.84 ^a^
EbS-3	0.411 ± 0.013 ^a^	43.9 ± 3.2 ^b^	0.73 ± 0.31 ^a^	6.66 ± 3.66 ^a^

* Values followed with different letters (a, b, c) for the same column are statistically significantly different (Tukey) at *p* < 0.05.

**Table 3:** Conductance, conductivity, ASR, and selectivity of hCEMs and CMX.

Sample	Conductance (mS)	Conductivity (mS·m^−1^)	ASR (Ω·cm^2^)	Selectivity (%)
CMX	46.05 ± 0.27 ^a^ *	8.42 ± 1.11 ^b^	2.81 ± 0.21 ^f^	91 ± 0 ^c^
UL5	42.76 ± 0.30 ^b^	12.96 ± 0.45 ^a^	4.19 ± 0.31 ^e^	63 ± 0 ^g^
UL6	42.54 ± 0.15 ^b^	11.96 ± 0.45 ^a^	5.13 ± 0.31 ^d^	74 ± 0 ^f^
UL7	42.09 ± 0.15 ^b^	11.43 ± 0.72 ^a^	6.37 ± 0.35 ^c^	81 ± 0 ^e^
EbS-1	38.54 ± 0.31 ^c^	8.54 ± 0.72 ^b^	6.75 ± 0.45 ^c^	89 ± 0 ^d^
EbS-2	38.36 ± 0.04 ^c^	7.18 ± 0.45 ^b^	8.49 ± 0.32 ^b^	92 ± 0 ^b^
EbS-3	33.56 ± 1.61 ^d^	4.55 ± 0.45 ^c^	10.53 ± 0.26 ^a^	95 ± 0 ^a^

* Values followed with different letters (a, b, c, d, e, f, g) for the same column are statistically significantly different (Tukey) at *p* < 0.05.

**Table 4:** Limiting current densities and voltages associated with the whole ED cell for each membrane.

Sample	Limiting Current Density (mA·cm^−2^)	Associated Voltage (V)
CMX	17.0 ± 4.3 ^a^ *	20.11 ± 2.03 ^a^
UL5	18.4 ± 0.9 ^a^	21.15 ± 2.55 ^a^
UL6	18.0 ± 0.3 ^a^	20.83 ± 2.56 ^a^
UL7	17.3 ± 3.6 ^a^	20.43 ± 0.83 ^a^
EbS-1	18.0 ± 1.6 ^a^	21.57 ± 0.49 ^a^
EbS-2	14.5 ± 0.7 ^a^	18.81 ± 1.58 ^a^
EbS-3	13.9 ± 1.5 ^a^	17.16 ± 0.31 ^a^

* Values followed with different letters for the same column are statistically significantly different (Tukey) at *p* < 0.05.

**Table 5:** Thickness and conductivity of hCEMs and CMX reported before and after electrodialysis.

Sample	Thickness before (mm)	Thickness after (mm)	Conductivity before (mS·cm^−1^)	Conductivity after (mS·cm^−1^)
CMX	0.144 ± 0.001 ^A^ **	0.152 ± 0.004 ^A^	8.42 ± 1.11 ^b,^*^,A^	5.90 ± 0.00 ^b,c,A^
UL5	0.421 ± 0.002 ^A^	0.427 ± 0.002 ^A^	12.96 ± 0.45 ^a,A^	11.70 ± 0.42 ^a,B^
UL6	0.406 ± 0.002 ^A^	0.406 ± 0.013 ^A^	11.96 ± 0.45 ^a,A^	10.25 ± 1.20 ^a,A^
UL7	0.389 ± 0.012 ^A^	0.393 ± 0.013 ^A^	11.43 ± 0.72 ^a,A^	10.15 ± 0.35 ^a,B^
EbS-1	0.462 ± 0.012 ^A^	0.465 ± 0.020 ^A^	8.54 ± 0.72 ^b,A^	10.6 ± 0.14 ^a,B^
EbS-2	0.423 ± 0.005 ^A^	0.438 ± 0.011 ^A^	7.18 ± 0.45 ^b,A^	7.4 ± 0.57 ^b,A^
EbS-3	0.428 ± 0.005 ^A^	0.407 ± 0.009 ^A^	4.55 ± 0.45 ^c,A^	4.65 ± 0.63 ^c,A^

* Values followed with different letters (a, b, c) for the same column are statistically significantly different (Tukey) at *p* < 0.05. ** Values followed with different letters for the same line (A, B) before and after are statistically significantly different (*t*-test) at *p* < 0.05.

**Table 6:** Energy consumption and current efficiency of all electrodialysis experiments using hIEMs or CMX.

Sample	Energy Consumption (Wh)	Current Efficiency (%)
CMX	13.31 ± 0.24 ^b,c,^*	44.28 ± 3.45 ^a^
UL5	14.16 ± 0.22 ^a,+^**	40.92 ± 1.90 ^a,b^
UL6	13.45 ± 0.51 ^a,b^	38.81 ± 7.00 ^a,b^
UL7	14.04 ± 0.19 ^a,b,+^	33.24 ± 2.53 ^b,+^
EbS-1	13.73 ± 0.19 ^a,b^	41.31 ± 2.06 ^a,b^
EbS-2	13.24 ± 0.18 ^c^	43.61 ± 4.28 ^a^
EbS-3	13.48 ± 0.19 ^a,b^	42.79 ± 3.56 ^a^

* Values followed with different letters (a, b, c) for the same column are statistically significantly different (Tukey) at *p* < 0.05. ** Values followed with + for the same column are statistically significantly different (Dunnett) than the reference (CMX).

**Table 7:** Performances of alternative CEMs on dairy product applications reported in the literature.

CEM (Manufacturer)	Solution	ED Unit	Duration (min)	Deminera- lization Rate (%)	Energy Consumption	Current Efficiency (%)	Reference
SC-1	WPC (10.0 wt%)	10 cell pairs 100 cm^2^/membrane	60	64.2	640 kWh/eq removed	84.2	Pérez et al. [35]
Neosepta CMX — homogeneous (Astom)	1.50 L nanofiltered whey (18.0–20.0 wt%)	8 cell pairs 37 cm^2^/membrane	260	90.0	26.5 kJ	70.0	Greiter et al. [45]
Neosepta CMB — homogeneous (Astom)	1.20 L acid whey (5.2 wt%)	2 cell pairs 36 cm^2^/membrane	180	90.0	0.014 kWh/g	80.0–90.0	Chen et al. [46]
Neosepta CMB — homogeneous (Astom)	2.00 L sweet whey (6.5 wt%)	2 cell pairs 36 cm^2^/membrane	180	75.0	5.9 kWh/ton of whey		Talebi et al. [47]
Neosepta CMX-fg—homogeneous (Astom)	2.00 L acid whey	2 cell pairs 100 cm^2^/membrane	180	67.0			Dufton et al. [48]
Neosepta CMX-fg—homogeneous (Astom)	0.35 L sweet whey (6.5 wt%)	2 cell pairs 10 cm^2^/membrane	210	70.0	9.375 kWh		Lemay et al. [39]
Ralex— heterogeneous (MEGA)	1.00 L sweet whey (7.0 wt%)	10 cell pairs 64 cm^2^/membrane	60	95.0			Diblíková et al. [49]
Ralex CMH-PES—heterogeneous (MEGA)	Sweet whey (5.5 wt%)	50 cell pairs 400 cm^2^/membrane	140–270	90.0			Šímonvá et al. [50]
CEM-PES—heterogeneous (MemBrain)	30.00 kg acid whey (20.0% wt%)	50 cell pairs 400 cm^2^/membrane	195	89.3	8.8 Wh/kg		Merkel et al. [51]
Ralex CM-PES TR I—heterogeneous (MEGA)	2.00 kg evaporated sweet whey (15.7 wt%)	10 cell pairs 64 cm^2^/membrane	180	98.0	4.4 Wh/kg		Nielsen et al. [52]

## Data Availability

Data is contained within the article.
